# Aortic Clamping Time Is Associated With Postoperative Thrombocytopenia Following Elective Open Abdominal Aortic Surgery

**DOI:** 10.1155/ijvm/5560285

**Published:** 2025-05-26

**Authors:** Victor Bilman, Jonathan Alk, Moshe Halak, Chen Speter, Ophira Salomon, Daniel Silverberg

**Affiliations:** ^1^The Department of Vascular Surgery, The Chaim Sheba Medical Center, Ramat Gan, Israel; ^2^Faculty of Medical & Health Sciences, Tel Aviv University, Tel Aviv, Israel; ^3^Thrombosis Unit, The Chaim Sheba Medical Center, Ramat Gan, Israel

**Keywords:** aneurysm, coagulopathy, open aortic surgery, platelet, thrombocytopenia

## Abstract

**Objective:** The objective of this study was to evaluate the incidence of thrombocytopenia following elective abdominal aortic surgery and identify the associated risk factors.

**Methods:** From 2009 to 2020, all consecutive patients undergoing elective open infrarenal abdominal aortic repair for aneurysms (AAAs) or aortic occlusive disease (AOD) were included in a prospectively maintained dedicated database and subsequently analyzed retrospectively. The perioperative dataset included the duration of surgery, aortic clamping time, graft configurations, blood loss, and blood products administered during the procedure. Univariate and multivariable analyses were conducted to identify risk factors for postoperative thrombocytopenia and assess its clinical consequences.

**Results:** A total of 100 patients (male *n* = 81, mean age of 68 ± 9.3 years) were included in the present study. The AAA group showed a higher prevalence of hypertension (*n* = 58 [76%]) versus AOD (*n* = 12 [50%]) with *p* = 0.014 and the use of vancomycin presurgery prophylaxis, *n* = 36 (47%) and *n* = 7 (29%), respectively, with *p* = 0.033. The AOD group presented a higher number of active smokers (*n* = 19 [79%]) versus AAA group (*n* = 30 [39%]) with *p* < 0.001. The overall mean operative aortic clamping time was 91.6 ± 35 min, with a significantly longer time in the AAA group (96.0 ± 36.1 min vs. 78.8 ± 28.5 min in the AOD group) with *p* = 0.046. The mean estimated blood loss was 1383 ± 834 mL, with a higher average of 1546 ± 878 mL in the AAA group versus 933 ± 472 mL in the AOD group with *p* = 0.002. A decrease in the platelet count was observed immediately after surgery, with a mean reduction from baseline of 40.5% ± 16.3% in the AAA, 41.9 ± 16.4% compared to 35.9% ± 15.4% in the AOD group with *p* = 0.553, reaching its nadir on postoperative Days 2 and 3. No major bleeding events associated with thrombocytopenia during the postoperative period were recorded. In 54 patients (54%), the platelet count returned to baseline by postoperative Day 5 (POD 5). Five patients exhibited a sustained platelet count drop of > 50% from baseline on POD 5 and were tested for heparin-induced thrombocytopenia, all of which returned negative results. On multivariable analysis, the patient age (OR 1.125; 95% CI: 1.024–1.236; *p* = 0.014) and clamping time (OR 1.034; 95% CI: 1.011–1.058; *p* = 0.004) were independently associated with a decrease in the platelet count.

**Conclusion:** Postoperative thrombocytopenia is common following an elective abdominal aortic surgery, but it was demonstrated that it typically resolves on its own by POD 5 without significant clinical consequences. The study identified the patient age, and aortic clamping time as independent risk factors for the development of thrombocytopenia. However, further research involving larger cohorts is needed to confirm these findings and better understand the underlying mechanisms and potential implications.

## 1. Introduction

Thrombocytopenia, defined as a platelet (PLT) count of <150 × 10^9^ L and any significant PLT decline, is a common phenomenon following major surgery [1, 2]. Severe postoperative thrombocytopenia (POTC) in the early postoperative period is highly correlated with increased morbidity and mortality [3]. Understanding the timing and severity of the POTC, along with the dynamics of the PLT count course, is essential for distinguishing normal POTC from pathological conditions. This differentiation is crucial for the effective management of this phenomenon and its underlying causes [1]. In the first postoperative days, POTC can primarily be attributed to hemodilution. However, consumptive and destructive causes, such as disseminated intravascular coagulation (DIC), drug-induced thrombocytopenia, immune thrombocytopenia, and posttransfusion purpura, are potential contributing factors [2].

The population of patients undergoing open abdominal aortic surgery, whether for aneurysms or occlusive disease, represents a distinct subgroup. Although both procedures involve aortic cross-clamping and reconstruction with prosthetic grafts, these patients are exposed to several significant risk factors that may contribute to the development of postoperative thrombotic complications [3, 4]. These risk factors include hemodynamic instability, aortic clamping, heparin exposure, increased blood loss, and the need for transfusions. Despite the procedural similarities, abdominal aortic aneurysm (AAA) and aortic occlusive disease (AOD) present unique clinical features that must be acknowledged. For instance, the large mural thrombus often seen in AAA patients may influence perioperative coagulation and fibrinolysis to a greater extent than in AOD patients [3–5]. Furthermore, AAA patients typically exhibit significantly lower PLT counts compared to those undergoing surgery for AOD, as shown by Bradbury et al. [3]. In contrast, AOD patients have been found to demonstrate higher baseline levels of thrombin–antithrombin III complexes, suggesting a state of chronic hypercoagulability [4]. Interestingly, despite these differences, postoperative changes in coagulation and fibrinolytic function following surgery for nonruptured AAA and AOD reconstruction do not differ significantly in terms of POTC incidence [3, 4]. While PLT consumption during surgery and its subsequent recovery over the following days has been well-documented, the underlying risk factors and clinical significance of this phenomenon remain poorly understood and warrant further investigation [4–6].

Although the association between thrombocytopenia, as part of the coagulopathy process, and poor outcomes in ruptured AAAs is well established, more up-to-date data are needed to better understand better POTC following elective open abdominal aortic surgery and its contributing risk factors [3]. This study is aimed at determining the incidence of postoperative thrombotic complications following elective abdominal aortic surgery for aneurysmal and occlusive disease, considering the distinct characteristics of each procedure, and identifying the associated risk factors.

## 2. Methods

### 2.1. Patient Population

Our study population comprised all consecutive patients who underwent elective open infrarenal abdominal aortic repair for aneurysms or occlusive disease at our tertiary hospital between 2009 and 2020. These patients were included in a prospectively maintained dedicated database and were retrospectively analyzed. Patients were divided into two groups: those with AAA disease and those with AOD. Data were collected from the patient's medical charts, including demographics, comorbidities, medications, and surgical indications. Intraoperative data included the duration of surgery, aortic clamping time, graft configurations, blood loss, and blood products administered during the surgery. Postoperative laboratory results were extracted from the patient's electronic medical records. Chronic kidney disease (CKD) was defined as an estimated glomerular filtration rate (eGFR) of less than 60 mL/min/1.73 m^2^, in accordance with international guidelines. Patients who underwent open repair for AAA or occlusive pathology in an urgent setting were excluded from this study. In all patients, only infrarenal aortic clamping was performed. Additionally, aneurysms with inflammatory or mycotic etiologies were also excluded. The Institutional Review Board of the Sheba Medical Center approved the study, and all patients gave written informed consent for anonymous data collection.

All patients were prescribed at least one anti-PLT medication prior to surgery, either aspirin 100 mg daily or clopidogrel 75 mg daily, which was continued postoperatively. No patient underwent surgery while on dual anti-PLT therapy. Additionally, all patients received a prophylactic dose of low molecular weight heparin (LMWH), specifically enoxaparin, which was administered until they achieved full mobilization. For patients requiring oral anticoagulant therapy, LMWH was administered at a therapeutic dosage throughout their hospital stay.

### 2.2. Preoperative and Postoperative Bloodwork

All patients underwent routine laboratory blood tests on the day of admission and daily thereafter, as needed, until discharge. These tests included a complete blood count (CBC), a chemistry panel (including liver and kidney function tests), and a coagulation profile (including prothrombin time (PT), activated partial thromboplastin time (aPTT), and fibrinogen levels). In cases where a patient's PLT count decreased by 50% or more from baseline, testing for heparin-induced thrombocytopenia (HIT) was conducted using immunoassays to detect antibodies. Functional assays were not performed.

### 2.3. Quantifying the Degree of Thrombocytopenia

Thrombocytopenia is defined as a PLT count below 150 × 10^9^/L. However, due to variations in patients' baseline PLT counts, we quantified thrombocytopenia as a percentage decrease from baseline rather than relying on an absolute PLT count. A reduction of more than 50% from baseline was categorized as severe thrombocytopenia.

### 2.4. Statistical Analysis

Descriptive characteristics are reported as mean ± standard deviations. Differences between groups (AAA vs. AOD) were tested using the chi-square test for dichotomous variables and the Student *t*-test for continuous variables. All tests were two-tailed, and a *p* value of ≤ 0.05 was considered statistically significant. Prior to conducting *t*-tests, the normality of continuous variables was assessed graphically using histograms. Variables that did not meet the assumption of normality were analyzed using nonparametric alternatives (e.g., the Mann–Whitney *U* test). Univariate analysis was conducted to identify categorical factors associated with a decrease in PLT levels using a chi-square test. A logistic regression model was conducted to evaluate clinical and sociodemographic variables that were either statistically significant in the univariate analysis (*p* < 0.05) or demonstrated a trend toward significance (*p* < 0.2), in order to identify potential risk factors associated with a reduction in PLT levels. The results are presented as the odds ratio with a 95% confidence interval. Statistical analysis was conducted using SPSS Version 25 software.

## 3. Results

### 3.1. Patient Characteristics

During the study period, a total of 100 patients (81% male) with a mean age of 68 ± 9.3 years underwent consecutive elective open infrarenal abdominal aortic surgery and were included in this study. The demographic details are summarized in Table 1. Among these patients, 76 underwent surgery for AAA and 24 for AOD. The AAA group was older than the AOD group (mean age: 70.5 ± 8.6 years vs. 60 ± 6.7 years, *p* < 0.001), with a higher incidence of hypertension (AAA *n* = 58 [76%] vs. AOD *n* = 12 [50%], *p* = 0.014). Diabetes mellitus (AAA *n* = 12 [16%] vs. AOD *n* = 9 [38%], *p* = 0.023) and current smokers (AAA *n* = 30 [39%] vs. AOD *n* = 19 [79%], *p* < 0.001) presented a higher prevalence in the AOD group.

The mean operative time was 208.0 ± 62.6 min, with an estimated median blood loss of 1383 ± 834 mL. Operative data is presented in Table 2. Clamping time (AAA 96.0 ± 36.1 min vs. AOD 78.8 ± 28.5 min, *p* = 0.046), estimated blood loss (AAA 1546 ± 878 mL vs. AOD 933 ± 472 mL, *p* = 0.002), transfused packed cells (AAA 947.1 ± 627.7 mL vs. AOD 477.3 ± 278.9 mL, *p* = 0.001), and transfused PLTs (AAA *n* = 8 [11%] vs. AOD *n* = 1 [4%], *p* = 0.031) were significantly higher in the AAA group.

### 3.2. Laboratory Analysis

Table 3 summarizes the preoperative and postoperative laboratory test results. No significant differences were observed between the AAA and the AOD groups regarding blood count, PT, aPTT, and fibrinogen levels.

### 3.3. PLT Changes and Surgical Outcome

**Figure 1:**
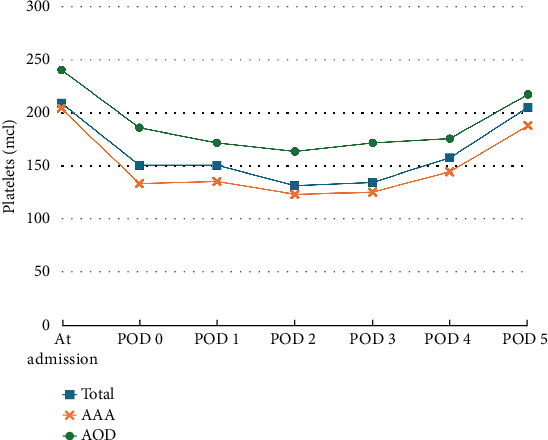
Trends in platelet count during admission. POD, postoperative day; AAA, abdominal aortic aneurysm disease; AOD, aortic occlusive disease.

Trends in the PLT count throughout the hospitalization are illustrated in Figure 1. A decrease in the PLT count was observed immediately after the surgery, reaching its lowest point on POD 2–3, with a mean reduction in the PLT count from baseline of 40.5% ± 16.3%. During the hospital stay, a gradual recovery in the PLT count was observed in all patients. By POD 5, the PLT count had returned to baseline levels in 44 patients. In 54 patients, the PLT count returned to baseline level at POD 5. No significant differences were observed between the group that returned to baseline PLT levels by POD 5 and those who did not, in terms of hemorrhagic complications and length of stay (LOS) (PLT baseline at the POD 5 group, 11.6 ± 6.7 days, vs. lower PLT at the POD 5, group 12.7 ± 9.2 days; *p* = 0.894).

Notably, no significant differences were observed in the intraoperative administration of packed red cells, PLTs, and plasma between the two groups. Postoperatively, no PLT transfusions were administered to any patients in the cohort, and no major bleeding events related to PLT count changes occurred during their hospital stay.

In 90% of the patients, the PLT count was within 30% of baseline upon discharge. Five patients exhibited a sustained PLT count drop in PLT exceeding 50% from baseline on POD 5 and were tested for HIT, with all results returning negative. A total of 23 patients experienced a PLT count decrease of more than 50% (AAA group: *n* = 20 [26.3%], AOD group: *n* = 3 [12.5%]; *p* = 0.161) and were classified as having POTC events for inclusion in the univariable and multivariable analyses (Table 4). On a multivariable analysis, patient age and clamping time were independently associated with a decrease in the PLT count. Each 1-year increase in age was associated with a 13% increase in the odds of POTC (OR 1.125; 95% CI: 1.024–1.236; *p* = 0.014), and each additional minute of clamping time increased the odds by 3.4% (OR 1.034; 95% CI: 1.011–1.058; *p* = 0.004) as shown in Table 4.

## 4. Discussion

In this study, we described the phenomenon of POTC following elective open aortic surgery and identified its contributing risk factors. POTC is a common occurrence among patients undergoing major surgery. When defined as a significant decline in the PLT count such as 20% or 30% reduction compared to preoperative baseline values, nearly all such patients experience this condition [2]. Our study identified aortic clamping duration during open abdominal aortic surgery as an independent factor influencing the severity of POTC. However, this finding did not correlate with increased morbidity and mortality.

The extent of POTC varies depending on the type of surgery performed. For example, Keles et al. [7] reported that 59% of cardiac surgery patients experienced significant POTC, compared to only 28% of hip surgery patients. This highlights the variability in PLT count decreases across different surgical procedures [7, 8]. These differences likely reflect varying degrees of hemodilution and PLT consumption. Regardless of the type of surgery, the nadir of PLT levels typically occurs on POD 2–3, followed by a gradual return to baseline by Day 5 [9, 10]. Griffin et al. analyzed thrombocytopenia following cardiopulmonary bypass (CPB) in 1364 patients and observed that PLT nadir tends to occur 48–72 h after surgery, with a mean PLT drop of 52.7% [9]. The authors identified that the use of CPB is a major risk factor for the development of POTC, which can lead to higher morbidity, including an increased risk of infection, development of acute kidney injury, and prolonged LOS [9]. However, this decrease in PLTs is rarely associated with bleeding complications and seldom necessitates PLT transfusion. These findings are consistent with the outcomes reported in the present study.

The causes of POTC appear to be multifactorial, making it challenging to isolate specific contributors. Several risk factors have been identified including the excessive fluid administration and transfusion of blood products [11]. Additionally, patient-specific factors such as age, comorbidities, functional status, and medications have been associated with POTC. In a report by Keles et al. [7], age over 80 years was associated with an increased risk of moderate-to-severe thrombocytopenia. Similarly, in our study, multivariable analysis revealed that advanced age was significantly correlated with a decline in the PLT count. Research further indicates that both PLT count and function decrease with age, likely due to age-related changes in the bone marrow [10, 11]. Hematopoietic tissue within the bone marrow and its cellularity remain relatively stable during middle age, but it declines significantly in individuals over 80 years old [12].

The cohort described in this study focuses specifically on patients who underwent aortic surgery. As such, all these patients required aortic clamping for a varying durations. The mechanism of thrombocytopenia due to aortic clamping is not fully understood. Bradbury et al. [3] described that aortic clamping leads to PLT sequestration and thrombocytopenia in the early postoperative phase, which may persist for many weeks. However, the clinical correlation between thrombocytopenia and morbidity or mortality remains to be clarified. The mechanisms underlying thrombocytopenia may be related to increased levels of circulating cytokines, particularly interleukin-6 (IL-6) [13, 14]. Other studies have found a relationship between aortic clamping time, neutrophil-derived leukotriene B4 production, arterial oxygen pressure, and tracheal intubation duration [15]. The PLT count may therefore reflect the confluence of events involving PLTs, leucocytes, and endothelial cell activation [15].

In a retrospective study involving 29 patients, Von Drygalski et al. examined a potential link between vancomycin use and immune thrombocytopenia, a condition where PLT counts decrease due to an immune response to the antibiotic [16]. One possible causes of thrombocytopenia in our cohort could be the prophylactic administration of vancomycin before surgery. However, due to the short duration of prophylactic exposure, it is unlikely that an immune-mediated response, such as immune thrombocytopenia, could have developed. Additionally, the fact that most cases of thrombocytopenia occurred on POD 2–3 further support our conclusion. Moreover, severe bleeding, a hallmark of vancomycin-induced immune thrombocytopenia, was not observed in any of our patients, further reducing the likelihood that vancomycin was the primary contributor to thrombocytopenia in our cohort [16].

Even though heparin is routinely administered during open aortic surgery, no cases of HIT were observed in this study. This suggests that HIT is not a common cause of decreased PLT levels in these patients. This observation aligns with the findings reported by other authors [7, 9, 17]. Griffin et al., in a retrospective study of more than 1300 patients undergoing CPB, reported that only 6 out of 356 cases of POTC were found to have HIT [9]. While HIT can be a potential cause of POTC, it should be considered a rare cause in the postoperative period. Type I HIT might contribute to mild and transient POTC due to intraoperative heparin administration. This nonimmunologic response to heparin treatment follows a similar trend observed in POTC, as it typically occurs within the first 48–72 h after the initiation of heparin and often resolves within 4–5 days once the heparin is discontinues [18]. However, no such association was demonstrated in the present study. Thus, screening for HIT in patients undergoing open abdominal aortic surgery should not be routinely performed for POTC unless clinically indicated.

Although open aortic surgeries for AAA and AOD are similar, they exhibit distinct clinical characteristics that warrant consideration. The present study identified some significant perioperative differences between the groups that may influence coagulation and fibrinolysis cascade, such aortic clamping time, estimated blood loss, and transfused packed cells. Pini et al. demonstrated in patients who underwent thoracoabdominal endovascular aortic repair that the presence of a large thrombus-free aortic lumen, which is only observed in AAA patients rather than in those with AOD, may have a more pronounced impact on perioperative coagulation and fibrinolysis [5]. Additionally, Bradbury et al. reported that patients with AAA generally have significantly lower PLT counts than those undergoing surgery for AOD, a trend that was also observed in the present study [3].

The natural course of POTC observed in this study appears to be benign, both in terms of PLT count, PLT recovery, and clinical outcomes. A nadir in the PLT count was observed 48–72 h after surgery, followed by a gradual increase toward baseline levels by POD 5. This pattern of self-limiting thrombocytopenia is consistent with findings reported in other studies [7–10]. During surgery, patients in both groups received large volumes of isotonic fluids and various blood products. PLT transfusions were administered intraoperatively to eight patients (10%) in the AAA group, compared to just one patient (4%) in the AOD group. Throughout hospitalization, as patients recover from surgery, PLT counts improved, likely due to increased bone marrow production or rebalancing of blood composition through the excretion of excess fluid via the urinary system. It is reasonable to assume that once POTC is identified, it will most likely resolve spontaneously over time without requiring specific intervention.

## 5. Limitations

This study has several limitations, primarily related to its retrospective design, relatively small sample size, and low event rate, which may constrain the robustness of the multivariable analysis. Additionally, patients with AAA and AOD were analyzed as a single cohort, despite representing distinct clinical entities. Although previous studies have not identified significant perioperative differences in coagulation or fibrinolysis between these groups, some evidence suggests that the extent of intramural thrombus in the aorta may influence the severity of postoperative thrombotic complications [3–5]. Larger, prospective studies are needed to further evaluate the predictors identified and validate the associations observed in this study.

## 6. Conclusions

POTC is common in patients undergoing elective open abdominal aortic surgery. The present study identified independent risk factors for POTC, including patient age and the duration of aortic clamping. This study demonstrates that POTC typically follows a benign course and is not associated with significant clinical complications in this patient population. Further research involving larger cohorts is needed to confirm these findings and better understand the underlying mechanisms and potential implications.

## Figures and Tables

**Table 1:** Demographics details and comorbidities.

**Variable** ^**a**^	**Total (** **n** = 100**)**	**AAA (** **n** = 76**)**	**AOD (** **n** = 24**)**	**p** ** value**
Gender (male)	81 (81%)	66 (86.8%)	15 (62.5%)	0.008
Mean age (years)	68 ± 9.3	70.5 ± 8.6	60 ± 6.7	< 0.001
Coronary artery disease	38 (38%)	30 (39.5%)	8 (33.3%)	0.589
Congestive heart failure	5 (5%)	4 (5.3%)	1 (4.2%)	0.820
Hypertension	70 (70%)	58 (76.3%)	12 (50%)	0.014
Diabetes mellitus	21 (21%)	12 (15.8%)	9 (37.5%)	0.023
Hyperlipidemia	68 (68%)	52 (68.4%)	16 (66.7%)	0.872
Current smoker	49 (49%)	30 (39.5%)	19 (79.2%)	< 0.001
Chronic obstructive disease	12 (12%)	10 (13.2%)	2 (8.3%)	0.526
Chronic kidney disease	12 (12%)	11 (14.5%)	1 (4.2%)	0.176
Vancomycin prophylaxis	42 (42%)	36 (47.4%)	7 (29.2%)	0.033

^a^Continuous data are presented as means ± standard deviation (SD); categorical data are given as counts (percentage).

**Table 2:** Intraoperative details.

**Variable** ^**a**^	**Total (** **n** = 100**)**	**AAA (** **n** = 76**)**	**AOD (** **n** = 24**)**	**p** ** value**
Tube graft	51 (51%)	57 (75.0%)	1 (4.2%)	< 0.001
Bifurcated, aortobi-iliac	19 (19%)	12 (15.8%)	0 (0%)	0.038
Bifurcated, aortobifemoral	30 (30%)	7 (9.2%)	23 (95.8%)	< 0.001
Operative time (min)	208.0 ± 62.6	208.3 ± 67.0	207.1 ± 49.5	0.934
Clamp time (min)	91.6 ± 35.0	96.0 ± 36.1	78.8 ± 28.5	0.046
Estimated blood loss (mL)	1383 ± 834	1546 ± 878	933 ± 472	0.002
Transfused packed cells (*n*)	85 (85%)	63 (82.9%)	22 (91.7%)	0.294
Transfused packed cells (mL)	825.5 ± 594.2	947.1 ± 627.7	477.3 ± 278.9	0.001
Transfused plasma	44 (44%)	38 (50.0%)	6 (25.0%)	0.343
Transfused plasma (mL)	572.9 ± 296.6	594.7 ± 309.3	435.0 ± 149.5	0.224
Transfused platelets	9 (9%)	8 (10.5%)	1 (4.2%)	0.031
Cell saver (mL)	459.2 ± 491.4	515.0 ± 547.6	275.0 ± 108.6	0.179
Length of stay (days)	11 ± 7	12 ± 8	11 ± 6	0.638

^a^Continuous data are presented as means ± standard deviation (SD); categorical data are given as counts (percentage).

**Table 3:** Preoperative and postoperative laboratory blood tests.

**Variable** ^**a**^	**Total (** **n** = 100**)**	**Abdominal aortic** **aneurysm (** **n** = 76**)**	**Aortic occlusive** **disease (** **n** = 24**)**	**p** ** value**
WBC, at admission (cells/*μ*L)	8.76 ± 3.4	8.71 ± 3.6	8.9 ± 2.2	0.564
WBC, POD 1 (cells/*μ*L)	12.1 ± 5.4	12.2 ± 5.8	11.9 ± 3.9	0.831
WBC, POD 5 (cells/*μ*L)	8.9 ± 3.3	8.9 ± 3.5	8.7 ± 2.6	0.418
Hb, at admission (g/dL)	13.3 ± 1.7	13.3 ± 1.7	13.4 ± 1.7	0.775
Hb, POD 1 (g/dL)	11.1 ± 1.2	11.2 ± 1.2	11.0 ± 1.5	0.577
Hb, POD 5 (g/dL)	10.9 ± 1.0	10.9 ± 1.0	11.1 ± 0.9	0.599
PLT, at admission (mcL)	225.1 ± 79.7	215.9 ± 75.3	259.1 ± 88.0	0.096
PLT, POD 0 (mcL)	176.3 ± 68.2	165.6 ± 61.8	211.6 ± 77.2	0.125
PLT, POD 1 (mcL)	159.9 ± 60.0	147.7 ± 51.6	197 ± 96.2	0.082
PLT, POD 5 (mcL)	210.6 ± 79.0	200.9 ± 70.9	242.4 ± 96.2	0.552
PLT decrease > 50% (*n*)	23 (23%)	20 (26.3%)	3 (12.5%)	0.161
Mean PLT decrease (%)	40.5 ± 16.3	41.9 ± 16.4	35.9 ± 15.4	0.553
MPV, at admission (fL)	8.7 ± 1.2	8.7 ± 1.0	8.5 ± 1.5	0.175
MPV, POD 1 (fL)	8.5 ± 1.1	8.6 ± 0.9	8.1 ± 1.2	0.066
MPV, POD 5 (fL)	8.2 ± 1.2	8.3 ± 1.1	7.7 ± 1.2	0.059
PT, at admission (s)	82.3 ± 14.4	82.1 ± 11.7	83.2 ± 20.9	0.189
PT, POD 1 (s)	80.8 ± 13.7	79.8 ± 13.1	84.3 ± 15.5	0.163
PTT, at admission (s)	31.3 ± 7.2	31.1 ± 7.2	31.9 ± 7.6	0.598
PTT, POD 1 (s)	28.9 ± 5.4	28.9 ± 5.2	28.6 ± 6.2	0.381
PTT, POD 5 (s)	28.5 ± 5.5	28.7 ± 5.8	27.3 ± 3.3	0.628
Fibrinogen, at admission (mg/dL)	240.9 ± 70.9	241.3 ± 68.2	238.7 ± 87.6	0.598
Fibrinogen, POD 1 (mg/dL)	337.9 ± 75.2	338.7 ± 72.7	331.5 ± 101.5	0.515

Abbreviations: aPTT, activated partial thromboplastin time; Hb, hemoglobin; MPV, mean platelet volume; PLT, platelet; POD, postoperative day; PT, prothrombin time; WBC, white blood cell.

^a^Continuous data are presented as means ± standard deviation (SD); categorical data are given as counts (percentage).

**Table 4:** Univariate and multivariable analyses of factors associated with platelet count decrease.

**Variable**	**Univariate analysis**			**Multivariable analysis**		
	**OR**	**95% CI**	**p** ** value**	**OR**	**95% CI**	**p** ** value**
Age + 1 (years)	1.098	1.027–1.173	0.006	1.125	1.024–1.236	0.014
Clamp time + 1 (min)	1.029	1.011–1.049	0.002	1.034	1.011–1.058	0.004
Coronary artery disease	2.704	1.043–7.009	0.041	1.788	0.492–6.491	0.377
Hypertension	2.255	0.687–7.401	0.180	1.844	0.444–7.653	0.400
Current tobacco use	0.51	0.189–1.375	0.183	0.723	0.188–2.777	0.636
Gender (male)	1.627	0.425–6.222	0.477			
Diabetes mellitus	1.6	0.531–4.821	0.404			
COPD	0.291	0.035–2.394	0.251			
Chronic kidney disease	1.222	0.299–4.99	0.780			
Tube graft	1.015	0.592–1.74	0.957			
Vancomycin prophylaxis	1.676	0.602–4.664	0.323			

Abbreviation: COPD, chronic obstructive pulmonary disease.

## Data Availability

The data that support the findings of this study are available on request from the corresponding author. The data are not publicly available due to privacy or ethical restrictions.
